# Burden and prevention of viral hepatitis in the Arctic region, Copenhagen, Denmark, 22–23 March 2012

**DOI:** 10.3402/ijch.v72i0.21163

**Published:** 2013-07-17

**Authors:** David FitzSimons, Brian McMahon, Greet Hendrickx, Alex Vorsters, Pierre Van Damme

**Affiliations:** 1 Documentation and Communication, Office of Governing Bodies, World Health Organization, Geneva, Switzerland; 2 Liver Disease and Hepatitis Program, Alaska Native Tribal Health Consortium, Anchorage, AK, USA; 3 Viral Hepatitis Prevention Board (VHPB), Centre for the Evaluation of Vaccination, Vaccine and Infectious Diseases Institute, University of Antwerp, Antwerp, Belgium

## Objectives

The Viral Hepatitis Prevention Board (VHPB), in collaboration with the WHO Regional Office for Europe, organized a meeting in Copenhagen in March 2012 on the burden and prevention of viral hepatitis in the Arctic regions, with the following aims: to provide an overview of surveillance systems for infectious diseases, to review the epidemiological situation and explain the high prevalence of viral hepatitis, to give an overview of the current prevention and control measures for viral hepatitis, to discuss the progress achieved in prevention, to review the possible implementation of new prevention strategies, control measures and monitoring systems, and to discuss the successes, issues and barriers to overcome, and the way forward. The focus of the discussions was the indigenous populations of the Arctic and subarctic regions. This report summarizes the background context, the health systems, surveillance and epidemiology of viral hepatitis in the region, the lessons learnt and matters for consideration, as well as possible future activities.

## Background context

The regions discussed embraced the lands north of the Arctic Circle (latitude 66°N) and subarctic territories north of latitude 60°N, covering all or northern parts of the United States of America (Alaska), Canada, Greenland, Iceland, Norway, Finland, Sweden and the Russian Federation. The populations consist of varying proportions of indigenous peoples (for instance, Alaska Natives, First Nations, Inuit, Sami and the “small peoples of the North”) intermixed with non-indigenous peoples. The latter includes people who had sought an alternative lifestyle, workers in the oil, gas and mining industries, and refugees and asylum seekers. Apart from Greenland and northern Canada, indigenous peoples generally form a minority of the populations. The indigenous peoples whose culture and traditions vary broadly speak many different languages.

Population densities are very low – about 4 million people live in this large and challenging area. There are no roads outside towns and travel is mostly by air or boat. Generally, socio-economic conditions are poor, with substandard housing, poor water supplies and sanitation, and limited employment opportunities; outside the few urban centres people live a subsistence existence, often facing food insecurity. Life-style diseases are widespread, for instance, drug use and alcohol abuse are common. Accidents are frequent, and suicide rates in indigenous young men are high. Considerable health disparities exist between these populations and those in the non-Arctic parts of the countries concerned, with higher infant mortality rates and lower life expectancy. Environmental challenges include “spectacular” climate change (1) and contamination of traditional foods.

Concern about the health and well-being of the populations of the Arctic and subarctic regions has resulted in international cooperation, exemplified by the International Union for Circumpolar Health, the Arctic Council (a ministerial forum) and the International Circumpolar Surveillance system (which does not yet, however, cover viral hepatitis). The EpiNorth project includes collection of data on viral hepatitis from 3 Nordic countries and the western part of the Russian Federation.

## Health systems

Hepatitis vaccination policies vary across the region and even within countries (2). Within the United States, Alaska Natives have received universal newborn, children and adult hepatitis B vaccination since 1984. The State of Alaska introduced a policy of universal hepatitis B vaccination of all newborns at no cost in 1994, expanding it to all children up to age of 18 years in 1997. Hepatitis A vaccine has been supplied at no cost by the State Government to all children aged 2–14 years since 1996, expanding coverage to 1–18-year olds in 2006. Since 2001, children have to produce a certificate of vaccination for admission into day-care facilities and school entry. In 2007–2010, the Centers for Disease Control and Prevention funded an adult hepatitis B vaccination initiative, but in 2012 all adult vaccinations (hepatitis A and B) were discontinued for budgetary reasons.

Greenland had followed Danish policy on hepatitis B vaccination until it became self-governing in 2009 whereupon it introduced universal hepatitis B vaccination of newborns as part of the childhood immunization program. The other countries or regions operate selective vaccination programs. In most administrations, the public sector provides the funds for hepatitis vaccines.

Different health care systems were described. In Alaska, about one-quarter of the population (more than 100,000 people) have no health insurance and, by law, hospitals cannot turn away uninsured patients. Publicly funded health services for specific groups (e.g. the Veterans Health Administration and the Alaska Native Tribal Health Consortium) co-exist alongside private medical practice. The Consortium, which is managed by Alaska Native tribal governments and their regional health organizations, provides comprehensive patient care through a network of village clinics and regional health centres, with active use of telemedicine and flying services for remote communities; in the main tertiary care hospital in Anchorage, traditional healers run a clinic beside western medical facilities. The health centres and village clinics are staffed by State-certified community-health workers.

The Canadian health care system is publicly funded, mostly free at point of use, with services provided by private entities. The Federal Government has traditionally undertaken regulation and standard setting and is legally obliged to transfer funds to provinces and territories, which have the responsibility for implementing health programs, such as the publicly funded immunization programs. Policies differ between provinces with no mechanism for coordination (3). In the Arctic and subarctic regions, most communities depend on air travel for delivery of supplies and transfer of patients to specialist health centres. Consequently, the annual health care costs in Nunavut and the Northwest Territories are about twice the Canadian average (nearly $5,200 per capita), even though telemedicine may play a mitigating role.

In Greenland, health care for the small population is entirely publicly funded, with free vaccination, treatment and medicines. The country has 5 health regions, each with a regional hospital; smaller communities are served by health centres and village clinics which are run by nurses or trained health assistants. Most communities are small, with no road links; transfer of patients and medical supplies, increasingly by plane or helicopter, accounts for 7% of the annual health budget. Regional centres and larger settlements have telemedicine services (although the need for staff training was highlighted).

Health systems in the Nordic countries are also mostly publicly funded, but with growing private sectors. Responsibility for health care is delegated, mainly to county councils in Denmark, Norway and Sweden and municipalities in Finland, with local politicians deciding on funding for vaccination programs. There are no special services for the indigenous Sami population. In all Nordic countries and Iceland, the hepatitis B vaccination programs target people in risk categories. Norway has the most extensive publicly funded policy, providing hepatitis B vaccination free to people in 10 categories, including immigrants from countries with high or intermediate prevalence rates of HBsAg carriage. Sweden alone provides vaccines free to prisoners and only in Finland are the household contacts of intravenous drug users vaccinated.

The Icelandic health care system is comprehensive. The State finances health care, with services being centralized. Most hospitals are run by the State and treatment is free. Primary health care is delivered through health care centres (which are both owned and run by the State) or by private practitioners who are partially paid by the patient.

Although basic health services, including vaccination (targeted to specified groups for hepatitis B), are provided free in the Russian Federation, treatment of hepatitis B and C is only partially funded (2), with the patients required to contribute to treatment and pay for viral load measurements, genotyping and some other tests (4). Reforms in the recent years, with large increases of funding for the health system, have enabled a massive increase in hepatitis B vaccination (see below). Apart from that, prevention of viral hepatitis in the country was described as being in need of improvement.

## Surveillance

In all of the countries, hepatitis A, B and C were reportable diseases, with reporting mostly based on clinical and laboratory data. In some countries, reporting led to follow-up. The value of reporting was enhanced when feedback was provided to those supplying the data, as in Canada. Generally, clinicians were described as the weak link in the reporting chain.

In Alaska, surveillance is a State responsibility. Laboratories and health providers are required by law to report cases of acute hepatitis A, B and all hepatitis C cases to the State. Since 2011, laboratory results can be submitted electronically. Funding is available for the follow-up of reported acute cases of hepatitis A and B, but no federal funding for follow-up of hepatitis C cases is available. The State laboratories offer testing for hepatitis C but stopped testing sera with positive anti-HCV results for PCR due to lack of funding. Chronic hepatitis B cases are currently not required to be reported but may be soon. All reports for hepatitis C are collated in 1 database (currently containing information on 14,000 cases) but no further enhanced surveillance has been undertaken and no differentiation is made between chronic or acute cases. The CDC's Arctic Investigations Program continues to collaborate with the Alaska Native Tribal Health Consortium and with the Division of Viral Hepatitis on viral hepatitis research and programs.

In Canada, the Enhanced Hepatitis Strain Surveillance System (introduced in 1998) provides clinical and laboratory data through sentinel surveillance of newly diagnosed cases of acute and chronic hepatitis B and C, with information on viral genotype and patient risk factors. The Northwest Territories joined the system in 2009. The legal prohibition of the breakdown of data on risk behaviours by ethnicity was circumvented by use of touch-screen technology that allows subjects to provide information anonymously and privately. That approach has generated valuable data for harm reduction in indigenous populations.

In Denmark, reporting of cases of chronic hepatitis B and C to the national clinical DANHEP database, established in 2002, is mandatory. Patients are identified with a unique coded Central Person Registry number and registered through the Internet. Data recorded include route of transmission, country of origin, test results for HBeAg and HBV DNA, viral genotype and treatment. By 2010, the database contained data on nearly 6,400 patients.

In Greenland, the National Board of Health collects surveillance data using the Danish unique coded personal identifier system. It has created several databases, including one on infectious diseases and childhood vaccination. Although cases of hepatitis are under-reported, these recent developments in data collection and handling augur well for generation of high-quality data and analyses.

In Iceland, active surveillance of defined populations for chronic hepatitis B and C covers immigrants from outside the European Union, pregnant women (but is incomplete for hepatitis B and controversial for hepatitis C), blood donors and intravenous drug users. Data from this active surveillance system are reported to the chief epidemiologist by health care centres, laboratories, hospitals and to a lesser extent, clinicians. Problems in distinguishing acute and chronic cases continue.

In the Nordic countries, surveillance data, based on laboratory and clinical reports with electronic notification, are not broken down by ethnicity (although data are collected on immigrants). Generally, the data on incidence are good but prevalence studies are limited, especially in risk groups. In Finland, electronic systems are well used for laboratory reporting (with automatic despatch of reminders to doctors) but only by very few treating physicians (3%). In Norway, immigrants are offered HBV and HCV testing within a few weeks of arrival. In Sweden, data with unique coded identifiers are collected at county and national levels. Generally, encrypted incidence data are publicly accessible through interactive websites as are data collected in the EpiNorth project.

Recent investments in the laboratory infrastructure and testing in the Russian Federation have improved the quality of surveillance data, which are based on clinical and laboratory reports and distinguish between chronic and acute cases. Testing for markers of HBV infection is obligatory for blood donors, pregnant women, patients with chronic liver disease and health care workers. Under-reporting exists.

## Epidemiology

### Hepatitis A

Based on the passive surveillance system installed in the region, subclinical infections are not reported. Moreover, clinically apparent hepatitis A disease has become rare in most of the region. Cases are seen mostly in returning travellers or are food-borne and imported, with some small outbreaks in men who have sex with men. Childhood vaccination programs have been extremely successful, reducing incidence rates in both children and adults. In Alaska (USA), for instance, by 2007 incidence rates in native populations were lower than the rates for other racial or ethnic groups in the United States. Similarly, since 2008 the rate in the Arkhangelsk region of the Russian Federation has been lower than that for the whole country. Protective immunity after vaccination persists for at least 17 years but may extend to 30 years (5, 6). Vaccination with 2 rather than 3 doses did not result in significantly different antibody titres. More evidence is needed for a decision about a one-dose schedule, but experience of its application during outbreaks indicates that it could be sufficient.

### Hepatitis B

Vaccination dramatically reduced incidence rates of acute cases in Alaska, Canada and the Russian Federation. The pioneering hepatitis B control program launched in Alaska in the early 1980s, the first designed to vaccinate all newborns, has resulted in long-term immunological protection of Alaska Native children for up to 30 years (7). Alaska now has the lowest rates of acute hepatitis B in the United States. In a cohort of vaccinated children (less than 20 years of age), no case of acute hepatitis B has been reported since 1992 and no case of hepatocellular carcinoma since 1999, indicating that universal newborn vaccination coupled with mass screening and immunization of susceptible Alaska Natives has eliminated HCC and acute symptomatic HBV infection among Alaska Native children (8). Currently, some 1,300 Alaska Natives are followed for chronic hepatitis B and an estimated 2,300–4,600 non-Native people are thought to be carriers of HBsAg.

In the Russian Federation (where by 2011 nearly half the population had been vaccinated, with excellent coverage rates for under-18-year olds), incidence rates of reported acute hepatitis B fell more than 27-fold between 1999 and 2010 in the Arctic regions. In 2011, only 5 cases were reported in the Arkhangelsk region (population of 1.13 million), where 13–15% of the population are tested each year for markers of HBV infection. Among 11 districts or regions, above-average rates were seen in only 2 (Chukotka and Khanty-Mansi). Rates of chronic hepatitis B were generally stable over the same period, at high levels in Kamchatka, Chukotka and Yamalo-Nenets. In the Arctic region, carriage of HBV (defined as being HBsAg positive without any clinical symptoms) fell nearly 6-fold. More cases were reported in the indigenous populations.

According to the data that were presented at the meeting, transmission of HBV through sexual contact remains the most common route in the Circumpolar region. However, extensive horizontal transmission (often occurring between the ages of 5 and 20 years) rather than from mother to child was reported from Greenland, a country with intermediate endemicity of HBsAg and an estimated 3,000–4,000 carriers of HBsAg. The prevalence data in the country indicate that only 17% of the population have never been exposed to hepatitis B virus (9).

Norway's selective vaccination program for intravenous drug users has not been very successful. Vaccine uptake was very low. As a result, outbreaks of acute hepatitis B are still seen in the north of the country.

Generally, the incidence of liver cirrhosis and hepatocellular carcinoma was lower than expected in Canada and Greenland but high in Alaska, although rates of hepatocellular carcinoma differed by Arctic population. In the Nordic countries, nearly all cases of chronic viral hepatitis B are seen in immigrants. Data from Denmark show that many cases of chronic hepatitis B are found in people of Asian or Middle Eastern origin.

Alaska Native people have 5 different genotypes of hepatitis B virus with genotype D predominating. Two subtypes of genotype D (D2 and D3) have been found in Alaska, while subtypes D3 and D4 were detected in Canada and a subtype midway between D1 and D2 was found in an outbreak in Greenland. In Iceland and the Russian Federation, genotypes D and A dominated, although in the north-eastern Russian Federation type C was common (26% of isolates) and some recombinant strains were also seen.

A new subgenotype B6 has been found in Alaska, Canada (extensively in the eastern Arctic region) and Greenland that appears to be less virulent than other HBV genotypes and is related to genotype B1 (Bj, originally found in Japan). It appears not to be a recombinant virus, in contrast to subtypes B2–B5. Subtype B6 has a higher mutation rate, and seems to have benign, or at least inapparent, consequences early after infection, but the fact that the viral DNA is integrated into the host cells means that the risk of hepatocellular carcinoma remains and it is too early to be sure that it really is benign.

Genotyping data illuminate the patterns of disease in different countries. Genotype distribution in Alaska corresponded closely to geography, and mapping clearly showed the migratory paths of genotype F throughout the south-west of the State. A theory was elaborated about the route of the possible introduction of hepatitis B viruses into Alaska from Asia around 10,000 BC and thence eastwards into other Arctic countries about 1,000 AD. However, although genotype F is found relatively frequently in Alaska, it is neither reported from Greenland nor found in Asia but is common in indigenous populations in South and Central America. Conversely, genotype B is more common in Greenland than in Alaska.

In Alaska, the association between the predominant route of transmission and genotype (10) was confirmed: genotype C was linked with maternal transmission. Hence, genotype F seems to be associated with a higher rate of hepatocellular carcinoma in children and adults. However, analysis of data from the DANHEP database showed that HBeAg status, not HBV genotype, predicted viral load in patients with hepatitis B in Denmark (11).

A community-based study performed in nearly 500 indigenous people in Canada revealed a high percentage (10%) of occult infections (defined as HBV DNA-positive and HBsAg-negative) (12). Recently, this study was repeated in a large group with more stringent diagnostics; 4% occult infection was recorded.

### Hepatitis C

Continued high prevalence rates of hepatitis C, related to drug use, are reported from the Russian Federation, Yukon (Canada), Iceland and northern Sweden. Outbreaks of acute hepatitis C occur in intravenous drug users in Norway, despite its extensive prevention programs (5 million needles are distributed to intravenous drug users each year – on a lesser scale, needles were being distributed extensively in small communities in the Yukon, Canada). In northern Sweden, recognition of the extent of the problem led to the introduction of innovative contact tracing and close liaison with concerned bodies in the community. Throughout Finland, hepatitis C presents a substantial burden of illness; the incidence in Lapland is similar to the national average. Although reported incidence rates of acute cases in the Russian Federation have fallen to low levels, the incidence of chronic cases has steadily increased over the past decade. In the Arkhangelsk region, about 13% of the population are tested annually for HCV infection. Chronic hepatitis C accounts for nearly three-quarters of all chronic hepatitis.

Data on hepatitis C in Alaska Natives are good, derived from screening programs which identify possibly half of those infected. State-wide in Alaska, the prevalence is similar to or may be slightly higher than rest of the United States. Possibly between 27,000 and 56,000 people have chronic hepatitis C virus infection in the State. Alcohol is a strong cofactor for adverse outcomes of hepatitis C; Alaska Natives with chronic hepatitis C are 17 times more likely to die of liver-related causes than people in the rest of the United States.

Annual incidence rates are slowly declining throughout Canada but rates are above the national average in the Northwest Territories, Nunavut and especially the Yukon. Hepatitis C was less prevalent in Inuit than in First Nations people (but spontaneous clearance of hepatitis C virus was common in the latter). Cases are mostly seen in older people, in men rather than women, and are linked with intravenous drug use. The situation in the Yukon may reflect more widespread testing and successful follow-up (including contact tracing), which would explain high rates seen for other diseases (e.g. sexually transmitted infections), but it may also be linked to the rise in drug use reported in Canada's northern regions. In contrast, hepatitis C was rare among the Inuit in Greenland and Denmark. The poor data for hepatitis C in Norway mean that no trend can be confirmed.

The predominant genotypes in Denmark, Iceland and the Russian Federation were genotypes 1 (principally 1b in the Russian Federation) and to a lesser extent genotype 3a.

In a national study in Denmark, treatment of patients with chronic hepatitis C with pegylated interferon and ribavirin was effective with sustained virological response particularly associated with infection with genotypes 2 and 3 (13). In Alaska, telepavir and boceprevir are additional drugs available for treatment. Triple therapy will be offered to patients infected with genotype 1 and moderate to severe fibrosis, whereas patients with genotype 2 and 3 HCV will be offered pegylated interferon and ribavirin irrespective of disease stage.

### Hepatitis D

As hepatitis D is not notifiable in most of the Arctic countries and regions, little or no information is available. Nevertheless, it continues to be a problem in Greenland where superinfection in young chronic carriers of HBV seems to have caused severe liver disease in HBeAg-positive children and an outbreak continues in one settlement. Hepatitis D virus has been reported in the Russian Federation as a major contributing factor to liver cirrhosis. Hepatitis D is not found in Alaska Natives who have chronic hepatitis B virus infection.

### Hepatitis E

In Norway, a few sporadic cases have been reported, most of which were imported. Generally, the few studies of anti-HEV prevalence show low rates, but in Chukotka (Russian Federation) antibody rates were twice as prevalent in non-indigenous people as in the native populations (14). About 3% of an isolated Inuit population in Canada were seropositive for anti-HEV antibodies with evidence of recent infection (15), but the source of the potential infection remains unclear – a different but limited study on potential zoonotic transmission found no evidence of hepatitis E virus in caribou.

## Lessons learnt

Overall, the indigenous Arctic populations have higher prevalence rates of chronic hepatitis B and some have higher rates of hepatitis C than other populations in those regions. Patterns of liver disease were the same in both populations. The presence of immigrants from highly endemic countries increases prevalence rates and may add to the high rates of viral hepatitis seen in Arctic regions. In countries with small indigenous populations, immigrants may also account for most chronic infections. For successful prevention strategies, the epidemiology and history of different populations need to be known.

Vaccination is widely accepted and dramatically reduces incidence rates, yet policies on hepatitis prevention and control differed both between and within countries, with universal childhood hepatitis B vaccination still not adopted by most administrations in Scandinavian countries. Vaccination coupled with improved water and sanitation and preventive measures (such as education of food handlers) have greatly reduced the incidence of hepatitis A, as in Alaska, but vaccination is recommended for travellers to areas where the disease remains endemic.

The impact of hepatitis B vaccination on incidence rates of acute hepatitis B and hepatocellular carcinoma add to the growing consensus that no vaccine booster dose is needed to maintain immunity (7). Programs that only target vaccination to risk groups are costly, and speakers concluded that this approach is not effective (16); altogether, universal childhood vaccination is the best and most effective policy. The information presented also showed that policies could be changed, with striking results. For instance, the limited vaccination strategy applied in Alaska and Canada 20 years ago and the Russian Federation until more recently (as in the Nordic countries today) had little impact; prevention and control only improved with the introduction of universal childhood and catch-up vaccination programs. Health economic analyses are essential for helping to change policies.

The implementation of a massive vaccination program in the recent years in the Russian Federation and its impact underline the effect of political will and commitment. Similarly, the achievements of public health workers throughout the region show what can be done by dedicated and motivated teams.

Telemedicine offers an effective means of improving access to care but needs investment in technology and training. Community medicine is highly developed, with primary care and liaison with larger centres provided by suitably trained community-health aide practitioners and nurses at community levels delivering good health care and liaison with larger centres, and with an increasing role of public–private partnerships.

Collections systems of data are varied, but in some countries, registries and databases offer valuable tools that can be applied broadly, including the follow-up of treatment of patients with chronic viral hepatitis, and can overcome confidentiality issues. The unique coded personal identifier is extremely useful. Feedback to the providers of data can enhance surveillance reporting and broaden disease prevention and control. Electronic reporting and notification systems are increasingly used but not applied everywhere. The ability to issue reminders (e.g. for α-fetoprotein testing in the screening of hepatocellular carcinoma in chronic hepatitis B in remote populations in Alaska) and to automate messaging adds to their usefulness and reduces the burden on staff. Overall, indigenous populations have similar prevalence rates, genotypes and risk factors for HCV infection as populations in the non-Arctic regions of countries, but hepatitis C places heavy burdens of disease in Canada and the Nordic countries, in particular in those territories where intravenous drug use is widespread. Every opportunity should be taken to screen at-risk individuals for anti-HCV antibodies and to provide people with chronic hepatitis C with advice about prevention of further infections and treatment possibilities. Prevention and education, particularly in relevant local languages, stay at the core of controlling viral hepatitis. Examples of successes were presented: reinvigorated contact-tracing programs demonstrated their worth in Sweden and innovative computer applications enhanced anonymous data collection in a Canadian province. Harm-reduction measures are often being practised on a massive scale, as with the distribution of needles to prevent transmission of hepatitis C between intravenous drug users in Norway, but their value has not been proven in this population.

## Matters for consideration

The enormous distances, extreme temperatures, low population densities among scattered communities, logistics of supply delivery (including avoidance of freezing of vaccine) and the costs of transporting specimens and patients all pose challenges that need innovative solutions. There were calls to expand circumpolar health cooperation, with fuller participation of countries and regions that are confronted with the same challenges, especially as hepatitis B and C continue to present serious problems.

Reporting systems need to be improved, with more comprehensive and up-to-date surveillance and better data on risk factors and routes of transmission in order to resolve the large number of cases whose origin of infection is categorized as “unknown”. Reporting by clinicians remains poor, despite incentives and rewards. Anonymous testing could be encouraged, especially in jurisdictions where reporting by name is required by law.

Despite reporting being a legal requirement, only limited funds are available for analysis of surveillance data and the application of the results for public health. Recently established databases could offer the means to document the performance of hepatitis B vaccination programs, especially given the likelihood of immunity after vaccination lasting for at least 30 years. In countries such as the Russian Federation where the whole population is being targeted by vaccination programs, some people with chronic hepatitis B will be vaccinated when there is no pre-vaccination testing. As a result, studies of the long-term efficiency of hepatitis B vaccination may be skewed by the presence of serological markers due to pre-existing infection that might otherwise be attributed to vaccine failure.

Issues of confidentiality need consideration. In Denmark, permission to use data in the DANHEP database has to be sought and approved by an ethics committee. Equity issues arise in relation to the provision of services for separate groups, including immigrants, where for instance free vaccination is not provided to the whole population.

The economic recession has had several consequences, including cessation of adult hepatitis B vaccination and HCV DNA testing in Alaska, and the drain on health budgets of the high costs of transporting patients and supplies. The introduction of new antiretrovirals for HCV treatment will further increase costs as more patients are treated. Funding for vaccination and treatment programs needs to be sustainable.

Ways to overcome the following main barriers to treatment of hepatitis need to be found: scattered populations living in small communities with poor access to hospital facilities and the need for blood samples to be transported by courier; lack of specialists; rapid turnover of hospital staff; patients’ lack of insurance; high drop-out rates; and lack of compliance.

Generally, country experts concluded that there was still room to improve screening of pregnant women for HBsAg. For hepatitis C, although the scientific relevance of screening pregnant women in Iceland for hepatitis C was contested, some speakers upheld it as an example of good public health practice, namely offering testing for treatable viral hepatitis infection to individuals who were otherwise difficult to reach when they present themselves for other medical reasons.

Raising awareness about viral hepatitis continues to be essential, for the young and old. Education materials should continue to draw attention to the persistence of the hepatitis B virus in the environment in order to convince parents about the value of vaccination of children before they reach the age of sexual activity. The consequences of life-style behaviour (for instance, excessive consumption of alcohol) in HCV and HBV transmission also need emphasizing. Also, continued efforts are needed to counter the misinformation circulated by anti-vaccine groups.

## Future activities

The quality of data (especially prevalence rates) needs improving, with more up-to-date and comprehensive surveillance, separation of statistics on acute and chronic cases, and analysis. The International Circumpolar Surveillance system could usefully extend its activities to cover viral hepatitis. Greater use should be made of registers and databases and electronic reporting mechanisms, including feedback to physicians and clinicians. Telemedicine needs to be extended and familiarity with its use ensured.

Preventive measures need reinforcing, including better infection control in personal-services settings, such as tattooing parlours. Multicultural and culturally sensitive education and training materials need further development. Advocacy is needed to raise support at policy-making levels for proven harm-reduction measures, including education about the dangers of drug use and its consequences.

Several aspects of prevention and control that need greater attention were identified (although the current financial constraints were recognized), including:

- continued surveillance and follow-up of subjects vaccinated against hepatitis A and B and focus on cohorts and the long-term public health impact on populations,
- evaluation of the recent universal HBV immunization program in Greenland and the possibly changing epidemiology of hepatitis B and D, and the role of hepatitis D, interaction with hepatitis B,
- economic data and analyses for policy development and decision-making, including the feasibility and economic attractiveness of the introduction of universal HBV vaccination in countries where this is not done,
- greater use of registry data for improving patient management, rates of vaccination and screening of close contacts, and for evaluating prevention of virus-related liver disease and death,
- evaluation of centralized treatment and the impact of treatment for hepatitis B and C on the incidence of hepatocellular carcinoma and cirrhosis,
- further investigation of immunological memory and duration of protection,
- reasons for non-response to hepatitis B vaccination and occult hepatitis B,
- identification and follow-up of HBsAg carriers,
- the natural history of infection with hepatitis B virus genotype B6,
- the possibly changing epidemiology of hepatitis C virus infection and drug use,
- guidance on protocols and regimens for prevention and treatment of hepatitis B, C and D.

Underlying all the above is the need for sustained commitment and political will; coordinated, comprehensive and nationally supported strategies on prevention, care and treatment; and sustained funding.
